# Developing a Bidirectional Academic–Community Partnership with an Appalachian-American Community for Environmental Health Research and Risk Communication

**DOI:** 10.1289/ehp.1003164

**Published:** 2011-06-16

**Authors:** Erin N. Haynes, Caroline Beidler, Richard Wittberg, Lisa Meloncon, Megan Parin, Elizabeth J. Kopras, Paul Succop, Kim N. Dietrich

**Affiliations:** 1 Department of Environmental Health, College of Medicine, University of Cincinnati, Cincinnati, Ohio, USA; 2 Neighbors for Clean Air, Marietta, Ohio, USA; 3 Mid-Ohio Valley Health Department, Parkersburg, West Virginia, USA; 4 Department of English and Comparative Literature, College of Arts and Sciences, University of Cincinnati, Cincinnati, Ohio, USA

**Keywords:** academic–community partnership, air quality, Appalachian American, community advisory board, community-based participatory research, manganese, risk perception

## Abstract

Background: Marietta, Ohio, is an Appalachian-American community whose residents have long struggled with understanding their exposure to airborne manganese (Mn). Although community engagement in research is strongly endorsed by the National Institutes of Health and the National Institute of Environmental Health Sciences in particular, little has been documented demonstrating how an academic–community partnership that implements the community-based participatory research (CBPR) principles can be created and mobilized for research.

Objectives: We created a bidirectional, academic–community partnership with an Appalachian-American community to *a*) identify the community’s thoughts and perceptions about local air quality, its effect on health, and the perception of risk communication sources and *b*) jointly develop and conduct environmental health research.

Methods: We formed a community advisory board (CAB), jointly conducted pilot research studies, and used the results to develop a community-driven research agenda.

Results: Persons in the community were “very concerned” to “concerned” about local air quality (91%) and perceived the air quality to have a direct impact on their health and on their children’s health (93% and 94%, respectively). The CAB identified the primary research question: “Does Mn affect the cognition and behavior of children?” Although the community members perceived research scientists as the most trusted and knowledgeable regarding risks from industrial emissions, they received very little risk information from research scientists.

Conclusions: Engaging a community in environmental health research from its onset enhanced the quality and relevance of the research investigation. The CBPR principles were a useful framework in building a strong academic–community partnership. Because of the current disconnect between communities and research scientists, academic researchers should consider working collaboratively with community-based risk communication sources.

Environmental health issues are prevalent and persistent in numerous communities across the United States, and particularly in disadvantaged communities. Participation by communities throughout the research process has been encouraged by the National Institute of Environmental Health Sciences (NIEHS), which recently initiated the Partnerships for Environmental Public Health (PEPH) umbrella program (Birnbaum 2009). The program goal of the PEPH is to engage communities in all stages of research, outreach, and educational activities to prevent, reduce, or eliminate environmental exposures that may lead to adverse health outcomes with particular emphasis on populations at highest risk. The program was founded based on the earlier reports from the NIEHS (O’Fallon and Dearry 2001; O’Fallon et al. 2003) that emphasized its role in supporting community-engaged environmental health research through community- based participatory research (CBPR) (Israel et al. 2001; Minkler and Wallerstein 2003; O’Fallon and Dearry 2002). CBPR is defined as “a methodology that promotes active community involvement in the processes that shape research and intervention strategies, as well as in the conduct of the research studies” (O’Fallon et al. 2000). The NIEHS endorses six guiding principles of CBPR: *a*) promote active collaboration and participation at every stage of research, *b*) foster colearning, *c*) ensure projects are community driven, *d*) ensure research and intervention strategies are culturally appropriate, *e*) define community as a unit of identity, and *f*) disseminate results in useful terms (O’Fallon and Dearry 2002).

The recognition that environmental health science research is enhanced through the implementation of the CBPR principles is gaining momentum (Brody et al. 2009; Brown et al. 2006; Minkler et al. 2008; Olden 1998; Quandt et al. 2001; Vásquez et al 2006; Wing et al. 2008); however, there remains a gap in the literature describing the implementation of CBPR principles in the process of developing a bidirectional academic–community partnership.

The purpose of this article is to document how the CBPR principles were used to build a partnership between an academic institution and a concerned community group in an Appalachian-American community. Here we describe how the partnership developed into a large-scale epidemiologic investigation that was driven by the concerns of the Appalachian community. We begin from the perspective of the community partner.

*Perspective from the community partner.* It’s 2:00 in the morning, and the second author (C.B.) is awakened by a bitter metallic taste in her mouth. She and her husband just completed construction of their dream home on a hilltop that overlooks the scenic Mid-Ohio River Valley in Marietta, Ohio. The bitter taste in her mouth was but one recurring symptom that she would experience, and Caroline was not alone. Other residents also experienced headaches, burning eyes, fatigue, muscle aches, tremors, and nose bleeds. The Mid-Ohio River Valley is also home to an industrial corridor, including a ferromanganese refinery—Eramet Marietta Industries Inc. (EMI; Marietta, OH). Until recently it was the only ferromanganese refinery in the United States, and it has released thousands of pounds of manganese (Mn) into the Marietta airshed [U.S. Environmental Protection Agency (EPA) 2009]. The > 50-year-old company leads the nation in fugitive airborne Mn emissions.

When driving past the refinery, the smell would force community members to roll up their windows, and they immediately turned on their windshield wipers to clear away the “toxic mist.” Concerned about air pollution, community members formed Neighbors for Clean Air (NCA), a nonprofit citizen action group. NCA mobilized to track smells by logging incidents in a “stink diary” and to record incidents of visible pollution with phone calls to each other. They also collected “swipe” samples from their homes and porches and paid for the metal analyses of these samples. In 2000, they wrote a letter to former Ohio Senator Mike DeWine, who petitioned the Agency for Toxic Substances and Disease Registry (ATSDR) to evaluate the health impacts from air pollution. Two years later, the ATSDR installed a total suspended particulate air monitor about 4.5 miles north/northwest of EMI, Inc., and in 2003 held a community meeting to assess the community’s concerns regarding air quality. Over the next several years, the ATSDR worked with the Ohio EPA to set up additional monitoring sites. The intermediate follow-up and reporting from these agencies left the community feeling frustrated with the slow progress and the lack of information to the community. During meetings with the federal agencies, the community was given information that was occasionally contradicted in other forums, so the community felt like “human subjects in an uncontrolled experiment.” In addition, tensions were growing between NCA and EMI, Inc. The community partner and other concerned community members had made many unsuccessful attempts to meet with EMI, Inc. management, who quickly dismissed their concerns and accused NCA of “pointing fingers.”

NCA’s determination to find answers to their questions about Mn exposure led them to seek help from Ohio Citizen Action (OCA). OCA is a nonpartisan grassroots consumer advocacy organization devoted to organizing and implementing community-driven campaigns that motivate major industries to take steps to reduce and prevent pollution emitted from their facilities (Ohio Citizen Action 2011). In June 2006, NCA, along with OCA, released a “Citizen’s Audit of Eramet Marietta” (Neighbors for Clean Air 2006) during a press conference held at the site of a newly installed ATSDR air monitor located in close proximity to the ferromanganese refinery. The Cincinnati-based OCA director at the time invited the first author (E.N.H.) to the press conference. OCA had accessed University of Cincinnati (UC) environmental health expertise previously, but this was the first contact E.N.H. had with that organization. During the press conference, she conversed with many community residents who expressed their numerous concerns and thoughts about their environmental exposure. As an academic, E.N.H. recognized the tremendous opportunity and assembled an academic research team to address the concerns of the community. The community offered to identify the health impacts, if any, of air Mn exposure on the neurodevelopment of children.

*Perspective from the academic researcher.* Manganese (Mn) is ubiquitous in the environment and occurs naturally in soil, air, water, and food. Unlike other metals, such as lead and mercury, Mn is an essential trace element required for normal growth and development, playing a key role in enzyme metabolism, immune function, bone growth, neurologic development, blood sugar metabolism, digestion, and reproduction (Aschner and Aschner 2005; Erikson and Aschner 2003). Mn deficiency is extremely rare in humans, but it has been suggested as an underlying factor in skeletal abnormalities and osteoporosis (Keen and Zidenberg-Cherr 1996) and seizure activity (Papavasiliou et al. 1979). Adequate intake of Mn is found in the diet, and the Food and Nutrition Board of the National Research Council has provided estimated safe and adequate daily dietary intake (ESADDI) levels for various groups. For instance, the ESADDI for infants and children ≤ 10 years of age is 0.3–3 mg/day, and adolescent and adult ESADDI levels are 2–5 mg/day (National Research Council 1989). Intake of dietary Mn is under strong homeostatic control (Papavasiliou et al. 1966), and toxicity associated with ingested Mn is rare; however, there have been reports of diminished intellectual function in children who have ingested drinking water with high Mn concentrations (Bouchard et al. 2007; Wasserman et al. 2006).

When inhaled, Mn can bypass the biliary excretion mechanism and directly enter the systemic circulation that results in neurotoxicity (Davis 1999; Keen et al. 1999). At high levels of exposure, Mn can produce a neurologic psychiatric disorder, manganism, which resembles Parkinsonian syndrome (Barceloux 1999; Lucchini et al. 2009), and has symptoms that can be clinically differentiated from Parkinson’s disease (Olanow 2004). These extrapyramidal and neuropsychiatric symptoms progress even after cessation of exposure (Pal et al. 1999). Adult studies have associated Mn exposure with deficits in neuropsychological and neuromotor function (Bowler et al. 2007; Mergler et al. 1999; Standridge et al. 2008) and increased risk of diagnosis of Parkinson’s disease (Finkelstein and Jerrett 2007; Lucchini et al. 2007). Low-level environmental exposure to Mn has been inversely associated with child cognition, behavior, and motor function (Riojas-Rodríguez et al. 2010; Rodriguez-Agudelo et al. 2006; Wright et al. 2006). Recently, the association between Mn exposure and adverse health outcomes among children has been identified as an inverted U-shaped curve. Henn et al. (2010) observed lower mental development scores among 12-month-old children with both high and low blood concentrations of Mn, and Zota et al. (2009) reported lower infant birth weights among infants whose mothers had both low and high blood Mn concentration.

Although a large-scale epidemiologic study of Mn exposure on children was warranted from a scientific perspective, the academic research team recognized the importance of understanding the views of the stakeholders before conducting an epidemiologic research study (Goldberg-Freeman et al. 2007), particularly if the research focused on a pediatric population in an Appalachian community. Although it is well documented that residents of rural areas are at risk for more health issues than are those living in urban areas (Hurley et al. 2000; Wewers et al. 2000), residents of Appalachia and other rural regions in the United States are some of the least researched populations (Elnick et al. 1995). Culturally, Appalachians are skeptical of outsiders and protect their personal privacy (Behringer et al. 2007), which is a potential barrier to researchers and others interested in improving the health of rural Americans. Residents of Appalachia and other rural regions in the United States have higher rates of poverty, lower education levels, and more limited access to health care (Friedell et al. 1998); compared with mortality rates of whites in the general U.S. population, Appalachian residents also have higher mortality rates for all causes of death (Halverson et al. 2004; Murray et al. 2005). Many Appalachian communities also bear an undue burden of environmental exposure to toxicants from coal mining (Hendryx and Ahern 2009), chemical industries (Steenland et al. 2009), metal refineries (Haynes et al. 2010), and environmental tobacco smoke (Wewers et al. 2000).

Thus, the academic research team from UC sought to engage an Appalachian-American community in a bidirectional relationship built upon the CBPR principles before developing a research proposal. A bidirectional relationship is defined as the movement or sharing of information between the community and the academic institution in a manner that is mutually beneficial. In this review, we describe how UC partnered with NCA to identify the primary research question and the community’s thoughts and perceptions regarding local air quality, perceived effects on health, and its perception of risk information sources. These partnership-building steps were essential for developing and conducting a large-scale epidemiologic research project and for gathering baseline information pertinent for designing community-appropriate risk communication messages.

## Materials and Methods

*Building an academic–community partnership.* The partnership between NCA and UC environmental health researchers was initiated by a key relationship between the first author (E.N.H.) and the second author (C.B.). Identifying a key community contact is not unusual in accessing a population of concern; in fact, it is a mechanism frequently used by other environmental health researchers (Brown 2003). As outlined by Brown (2003), academic–community relationships require that the members of the research team have a sincere interest in the community and its research agenda and that they become well acquainted with the history of the community’s struggle with its environmental issue. Because UC is approximately 3.5 hr from Marietta, Ohio, the academic research team and community members engaged in follow-up conversations by phone and e-mail. The conversations involved sharing information between the community and the academic institution. The NCA expressed its concern about the community’s exposure, previous experiences with the ATSDR, U.S. EPA, and Ohio EPA, and its desire for a health study. At an initial meeting with the NCA, E.N.H. discussed the known health impacts of Mn exposure. This information sharing laid the foundation of the partnership and demonstrated a mutual respect for each member’s expertise, thus applying CBPR principles by acknowledging the community as a unit of identity and steering the potential research agenda onto the issues most relevant for the community.

*Forming a community advisory board.* The next step in building an academic–community partnership was forming a community advisory board (CAB). The NCA director and other members assisted in identifying key members of the community to form a CAB. Each individual was contacted by E.N.H. and invited to participate. The first CAB meeting was organized by the NCA director and held in a neutral location within the community—a meeting room in a church in Marietta, Ohio. The initial CAB meeting was attended by 28 local residents whose occupations included homemaker, carpenter, industry representative, health department official, and a local college professor. Each attendee signed a UC Social and Behavioral Institutional Review Board (IRB) consent form to participate in the focus group. After introductions, the academic research team initiated the meeting by providing a summary of the current state of the science regarding the known health effects of Mn exposure, primarily from studies conducted on occupationally exposed adults. An active dialogue then ensued, and the CAB posed the following question: “What questions do you have about air quality in your community?” A member of the academic research team recorded responses on a flip chart.

The CAB identified a myriad of research questions, including the following:

Does Mn affect cognitive development of children?
Does Mn affect behavior and school performance in children?
Does Mn affect the health of seniors?
Where is Mn found in the environment (food, water, dairy products, meat)?
What is the safe level of exposure?
How does time spent outdoors and exercise affect dose?
Is Mn exposure related to health problems such as respiratory diseases, autoimmune diseases, blood disorders, and cancer?

Although the CAB was composed of a diverse group of residents, the CAB had a unified vision: to understand their exposure. Using consensus decision making, we identified the primary research question of the CAB: “Does Mn affect the cognition and behavior of children?” The academic research team communicated to the CAB the importance of collecting preliminary data if a proposed large-scale epidemiologic study was going to be developed for the community. Throughout our initial meetings and discussions, we applied the CBPR principles of fostering a colearning environment and began building the research capacity of each partner.

*Jointly conducting pilot research*. We planned two unique pilot studies: a community-wide survey and a pilot research study. The pilot study would be useful to determine the suitability for an epidemiologic study in the community and to ascertain levels of Mn in biological specimens, and a research survey would validate the concerns presented by the CAB and determine if the CAB does in fact represent the views of the community at large. The CAB played a key role in developing and executing both of these preliminary studies.

Together the academic research team, community partners, and the director of OCA, with input from NCA members, developed a survey instrument for community-wide distribution. The academic research team and the NCA community partner informally interviewed CAB members and experts in environmental health research to determine the questions to be asked and to identify potential responses for closed-ended questions. The team identified standard survey instruments for these topics when possible. The initial draft of the survey was developed by the academic research team and distributed to the community partners for their input. During the first phone conference with the community partners regarding the survey draft, the academic research team realized that the draft survey was not addressing community needs, so multiple revisions were made to the survey. The community partners provided insight into their community, providing new survey questions and community-appropriate multiple-choice responses. A specific example is the question pertaining to barriers faced by the community in improving the community’s air. The community partners provided all potential responses. Thus, the final version of the survey was a result of multiple phone conversations and e-mails between the academic research team and community partners, with continued feedback from OCA and NCA members and UC environmental health researchers.

To echo the CAB’s interest in research, the survey asked respondents the following question: “If a research study was conducted in your community related to air quality, what health outcomes do you think would be most important to study?” They were provided a list of seven possible health outcomes and an option to write in “other.”

In order to assess the risk communication channels appropriate for the Marietta-area community, respondents were queried about their use of mass media and interpersonal sources of information about risks related to industrial emissions in their community. Our goal was to determine their information-seeking behavior, including information sources related to local industrial emissions, their level of trust in these information sources, and how they rate the level of knowledge from each information source. We used a modified version of the questionnaire created by McCallum et al. (1991). We asked respondents to rate their perception of the amount of information received, knowledge, and trust regarding local industrial emissions for the following sources: news reporters, friends and neighbors, people you know who work for a local industry, local government officials, state government officials, chemical industry officials, medical doctors, research scientists, teachers, Web sites, environmental groups, town meetings, church meetings and announcements, and other. Possible responses were “a lot,” “some,” “hardly any,” or “none.” A draft of the survey instrument was reviewed by environmental health experts, the CAB, and OCA and then piloted on several NCA members to test the completeness and readability of the survey instrument.

The CAB identified the appropriate channels to distribute recruitment information. We distributed announcements to newspapers and radio stations and made paper copies available at key locations, including local grocery stores, libraries, colleges, and community buildings. The questionnaire was made available online using a Web-based survey tool (SurveyMonkey®, Palo Alto, CA). In addition, we mailed approximately 250 surveys with return postage to individuals provided in a directory of NCA contacts. The information collected from this questionnaire was anonymous and was approved by the UC Social and Behavioral IRB. The survey was available over a 5-month period, from June through October 2007, throughout Washington County; Marietta is the county seat of Washington County.

The methods for conducting the pilot research study are described elsewhere (Haynes et al. 2010). The CAB was strongly instrumental in the conduct of the pilot research study. CAB members helped identify potential locations for the study and recruitment methods, thereby building on the strengths and resources of the community in a collaborative research process. Their involvement resulted in the development of the Community Profile Survey (CPS), used to identify potential study participants. As described by Haynes et al. (2010), the CPS collected demographic information including age, years lived in Marietta, number of children, occupation, former residential addresses, and length of time lived at those former residences. The CAB members also distributed the CPS throughout the community. Given the NCA director’s high profile within the community, potential participants also called her home to inquire about the research study and complete the CPS over the phone. During the pilot, interest about the study spread through the community by word of mouth, and potential participants arrived during the study-testing days. These walk-ins completed the CPS and were screened for eligibility. All participants signed a UC IRB approved consent or assent form before data collection. The results of the pilot study have been reported previously (Haynes et al. 2010; Standridge et al. 2008).

## Results of the Community-wide Survey

The survey was completed by 271 respondents. We excluded individuals who returned a survey and reported living outside of Washington County (*n* = 42). Thus, 229 respondent results were analyzed.

*Community concern about local air quality.* The overwhelming majority of survey respondents were “very concerned” to “concerned” about the quality of the air (91%), quality of the water (85%), and quality of the soil (74%) as opposed to reporting “slight concern” to “no concern” about air quality (5%), water quality (7%), and soil quality (5%; Table 1). A high percentage of survey respondents were also “concerned” to “very concerned” about the odor in the local air (75%) compared with only 12% who were reportedly “slightly concerned” to “not concerned” about the odor in the community’s air. Additionally, 77% (*n* = 175) were “concerned” to “very concerned” about Mn exposure in their community as opposed to only 27 respondents (12%) who were not concerned.

**Table 1:** Respondents’ concern about local environment, odor, and Mn exposure (*n* = 227–229).

Local environmental concern	Very concerned to concerned	Neutral	Slightly to not concerned
Air	209 (91)	8 (3)	12 (5)
Water	193 (85)	19 (8)	15 (7)
Soil	168 (74)	33 (15)	25 (5)
Odor (smell) in air	172 (75)	30 (13)	26 (12)
Mn exposure	175 (77)	25 (11)	27 (12)
Data represent number of respondents (%).			

**Figure 1:**
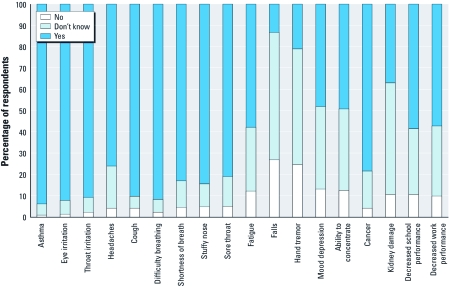
Health symptoms or conditions perceived to become worse when the quality of air is worse (percentage of survey respondents indicating each symptom or condition).

*Risk perception.* The community respondents perceived air quality to influence their health (93%) and the health of their children (94%; Table 2). They also perceived the amount of Mn to affect their health (71%) and the health of their children (73%). In addition, they also reported an association of symptoms with the quality of air (Figure 1). We asked them if they thought particular health symptoms or conditions became worse when the quality of air became worse. The vast majority (≥ 75%) perceived health symptoms (asthma, throat irritation, headaches, difficulty breathing, stuffy nose, shortness of breath, sore throat, cough, eye irritation, and cancer) became worse when the quality of air became worse (Figure 1). Other symptoms strongly perceived to be related to air quality included mood/depression, fatigue, decreased school performance in children, and decreased work performance (Figure 1). The community was predominantly “not sure” if falls, hand tremor, and kidney damage were related to air pollution. When asked if Mn specifically contributed to health symptoms, > 57% of all respondents were “unsure” if the amount of Mn in the air affected their health or their children’s health (data not shown).

**Table 2:** Air quality risk perception and willingness to participate in a research study (*n* = 189–229).

Respondents’ perception of risk	Extremely likely to likely	Not sure	Unlikely to extremely unlikely
Air quality affects your health		212 (93)		7 (3)		10 (4)
Amount of Mn in the air affects your health		163 (71)		55 (24)		11 (4)
Likelihood of participating in a research study		175 (77)		41 (18)		12 (5)
Air quality affects your child’s health		214 (94)		5 (3)		9 (4)
Amount of Mn in the air affects your child’s health		167 (73)		52 (23)		10 (4)
Likelihood of allowing your child to participate in a research study		120 (63)		41 (22)		28 (15)
Data represent number of respondents (%).						

Survey participants were asked to rate their level of understanding of Mn exposure in their community. Only 11% reported “a lot,” whereas 22% reported “no” understanding. When asked if they wanted to learn more about their potential exposure to Mn, 87% reported “yes,” nine (4%) reported “no,” and 8% reported “don’t know” (data not shown).

*Barriers to improving air quality.* The survey participants were asked to identify the barriers they believed their community faced to improve their local air quality. They could select all options that applied or respond “other.” On average, each respondent selected four of the options. The leading barriers perceived in the community included “fear of impact on economy” (88%, 197 of 224), lack of enforcement from federal government (77%, 173 of 224), lack of research related to quality of air and health (63%, 142 of 224), and lack of enforcement from local government (59%, 132 of 224; data not shown). Twenty-two percent (50 of 224) responded “other,” some of which could be categorized into the choices given (lack of enforcement from governmental agencies and economic impacts), but others were unique, including lack of community education on the topic of air quality (16%, 8 of 50), failure of media to recognize or draw attention to the problem (6%, 3 of 50), and perceived “helplessness,” “apathy,” “malaise,” and “good old boy government.”

*Community-identified research areas.* The leading health outcome of interest was “cancer” (data not shown). A total of 13% (30 of 229) of the respondents entered a health outcome in the “other” category. Seventy-seven percent (177 of 229) of all survey respondents selected cancer as an important health outcome to study. Other leading health outcomes included “overall health” (66%, 152 of 229), asthma (56%, 128 of 229), and allergies (52%, 120 of 229). Interestingly, 33% (76 of 229) of all respondents selected “brain development.” Within the “other” category, approximately 40% of all responses can be equally divided into “immune disorders” and “breathing problems.” Other responses included birth defects, sarcoidosis, cardiovascular disease, and headache. One respondent stated, “You have to study it all; we don’t want to be ill because of the air pollution.”

**Figure 2:**
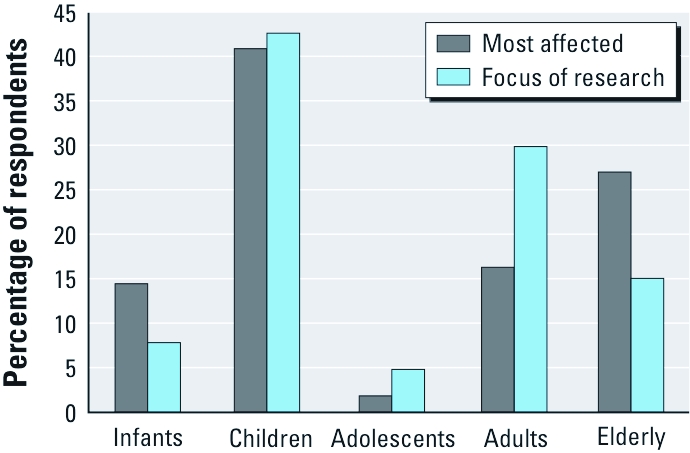
Community-identified populations that are most affected by air quality and on whom the research should focus (percentage of survey respondents indicating each population).

Respondents were asked to identify which group of people they thought was most affected by the quality of air in their community and, if a research study was conducted, on whom the research should focus. Respondents identified children as the leading group most affected by air quality and on whom research should focus (Figure 2). The respondents viewed the elderly as the second leading group most affected by air quality, followed by adults and infants. Interestingly, respondents did not view adolescents as a group affected by air quality or as a group on whom research should focus.

*Willingness to participate in research studies.* We also asked the participants’ likelihood to participate in a research study. The vast majority of respondents reported that they would be extremely likely and likely to participate (77%, 175 of 228), whereas 18% (41 of 228) were unsure (data not shown). Most the respondents would also allow their children to participate (63%, 120 of 189), whereas about a quarter of the respondents (22%, 41 of 189) were unsure and 15% (28 of 189) reported that they would be unlikely or extremely unlikely to allow their child to participate in a research study.

**Figure 3:**
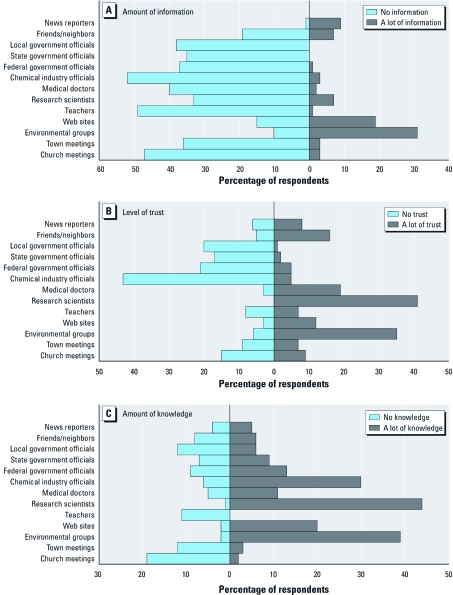
Community-identified amount of information received (*A*), perceived level of trust (*B*), and amount of knowledge (*C*) for each information source on risk of local industrial emissions (percentage indicating “no”and “a lot”).

*Community perception of information sources.* Respondents were asked where they would search for information about the risks of local industrial emissions in their community’s environment. The response choices were “yes” or “no,” and the questions were answered by 226 respondents. Web sites (88%, 161 of 183), environmental groups (88%, 166 of 189), news reporters (84%, 152 of 182), research scientists (83%, 121 of 145), medical doctors (73%, 103 of 141), and town meetings (62%, 82 of 133) were the leading sources of their searched information (data not shown). When asked to rate the amount of information received about the risks of local industrial emissions in their environment, the leading information sources, those selected as “a lot” of information received were environmental groups (31%, 68 of 216) and Web sites (19%, 38 of 204; Figure 3A). The most highly trusted sources of information (percentage of respondents indicating “a lot of trust”) were research scientists (41%, 87 of 212), environmental groups (35%, 75 of 217), medical doctors (19%, 39 of 209), and friends/neighbors (16%, 35 of 213; Figure 3B). Participants also perceived research scientists as having the highest (“a lot” rating) level of knowledge (44%, 93 of 211) of local industrial emissions, followed by environmental groups (39%, 84 of 217) and chemical industry officials (30%, 62 of 210; Figure 3C). As evident in Figure 3A, respondents identified almost all categories, except news reporters, environmental groups, and Web sites, as sources from which they receive “no” information. Survey respondents have “no” trust for the information received from officials from the chemical industry (43%, 90 of 208); local (20%, 43 of 212), state (17%, 37 of 213), and federal government (20%, 44 of 214); people they know who work for local industry (16%, 34 of 211); and church meetings and/or announcements (15%, 30 of 204; Figure 3B).

*Codevelopment of grant applications.* After the pilot studies, we began planning two NIEHS grant applications: a CBPR award and Partners in Research (PIR) award. The CBPR grant would focus on scientifically addressing the community’s primary research question related to the neurobehavioral effects of Mn on children, and the PIR grant would focus on furthering the academic–community reciprocal research relationship and build on the risk communication findings from the community-wide survey.

The community partners were very involved in the planning process. Multiple in-person meetings were held with the academic research team and community partners in Marietta to discuss what a large-scale study would look like and what resources the community offered that could be used for the study. Although the academic research team and UC environmental health researchers developed the hypothesis, specific aims, and research methodology for the grant application, the input of the community, in terms of what would be feasible and appropriate, was extremely important in the development of the application. For instance, two CAB members are Marietta College faculty members who provided a convenient study location and oversight of undergraduate and graduate students participating in data collection. Other resources in the community included networks with media outlets to engage the community in the ongoing study through local media outlets and subject recruitment ideas. The community partners were also involved in identifying research questions. For instance, the CAB and community partners were very concerned about Mn exposure through ingestion of locally grown and raised food products, such as fruits and vegetables, milk and beef. To address this, potential local exposure sources have been incorporated into a nutritional questionnaire developed by an expert in nutrition science.

Involvement of the community partners was also very important during the development of the R03 proposal, which evaluates the effects of providing reciprocal environmental health education for local media, health care providers, UC journalism and technical writing students, and community members. The community partners were instrumental in identifying the target populations and in determining the education topics and formats for each group. The community’s role in planning the research was critical in implementing CBPR principles and helped to ensure that the research agenda would be community driven and that the results would be disseminated in culturally appropriate, accessible, and useful formats.

## Discussion

Through the development and conduct of the pilot research study, we were able to identify the assets of the community for an epidemiologic study and determine the feasibility of a large-scale research project in partnership with the community. We were also able to build the capacity of the community for research through their participation in planning and conducting the core epidemiologic study. Data were presented to the CAB for their interpretation of the findings and discussion of how, when, and where to share the findings with the community-at-large.

The relationship between the community and university researchers was strengthened through their collaboration on the survey research project. The CAB helped build the questionnaire, including identifying  community-anticipated responses to closed-ended questions. The initial draft of the questionnaire developed by the university team is a shadow of the final survey product. The input from the community dramatically changed the survey questions and possible responses. Through both of these jointly conducted research efforts, mutual trust and respect for each other’s expertise grew. As a result of the multiple meetings held in the community and university campus, along with attending several community events, the academic research team researcher and her investigative team became part of the Marietta community, and the NCA director and other CAB members became an essential part of the university research effort (the outcome of a productive, bidirectional relationship). The survey also provided an outlet for community members to voice their thoughts and perceptions about local air quality and their interest and willingness to participate in a research study before the development of a research proposal. This opened the dialogue between the community and the academic institution, thereby heightening the quality of the research.

The CAB’s views and concerns about air quality were echoed in the community-wide survey. The survey respondents, CAB, and research community were in agreement that young children and the elderly are highly susceptible, at-risk populations for exposure to air pollution and are the populations on which research should focus (Hong et al. 2007; Saric and Piasek 2000; Tasker et al. 2003). Although not trained in environmental health science, community members demonstrated their native understanding of the problems they faced because they were able to identify at-risk populations that warrant further research. Their perception of air-quality–related health outcomes predominantly included those related to respiratory function. These community perceptions match scientific findings of positive associations between airborne particulate matter and pulmonary health effects. Their uncertainty of the relationship between Mn and health effects is also accurate; scientifically, little research has been conducted on the potential harmful effects of Mn on other organ systems, including the lungs (Barceloux 1999; Roth and Garrick 2003). Further, this evidence that community members’ native understanding of environmental health problems coincide with scientific evidence supports the concept that community members should be an integral part of the initial phases of the research process, including the determination of the research study questions and design.

Survey respondents reported their concern about the quality of air (91% concerned to very concerned), and they perceived the air to have a direct impact on their and their children’s health (93% and 94%, respectively). This finding supports the claim by Elliott et al. (1999) that the “relationships between an environmental exposure (e.g., air pollution) and health (e.g., respiratory effects) are mediated by perceptions of the ‘exposure’ (e.g., air quality), which are in turn influenced by a host of both individual and contextual factors.” Of the respondents, > 90% also could identify specific health effects they perceived to be strongly linked to air quality (asthma, cough, eye irritation, throat irritation, and difficulty breathing). This finding of a relationship between people’s risk perceptions and air quality, especially when people have illnesses that could be caused by pollution (e.g., respiratory problems), is not new (Bickerstaff and Walker 2001; Elliott et al. 1999; El-Zein et al. 2006). Because respondents were able to identify related consequences of poor air, we wanted to know how they identified poor air. One way people make risk perception judgments is through their personal or sensory encounters or, as termed by Scammell et al. (2009), their use of “tangible evidence.” More than 70% of the respondents listed the smell and sight of the air as ways they perceived poor air quality.

The community’s identification of cancer as a leading community health concern (80%) is not surprising because cancer mortality in Appalachia is significantly higher than for the U.S. as a whole (Centers for Disease Control and Prevention 2002; Halverson et al. 2004; Wingo et al. 2008). This concern about cancer was also mirrored in the CAB, but not to the same degree as in the survey. One likely reason for this difference is that the academic research team discussed the known health effects associated with Mn exposure with the CAB on multiple occasions (meetings, phone conversations). The information shared included that although Mn is known to cause DNA damage, it does not pose a significant carcinogenic risk (Desoize 2003), thus reducing, but not eliminating their concern about cancer. It should also be noted that Washington County had a higher cancer incidence rate for all sites and types combined compared with the U.S. population from 2001 through 2005 (509.3 vs. 467.4 per 100,000, respectively) (Ohio Department of Health 2008), so fear of cancer in the community is a valid concern. During the consensus process of selecting the primary research question, we discussed that although all research questions presented were valid, they could not all be addressed in a single epidemiologic study and that further studies would be needed to address each research idea.

Through the community-wide survey, the community was able to express their interest in participating in a research study. The vast majority of participants reported being likely or extremely likely to participate in a research study (≥ 77%) and to allow their child to participate in a research study (≥ 63%). We did not further investigate the reasons for their likeliness to participate; however, given their high level of concern about Mn and air quality and their perception that the air quality has a direct impact on their and their children’s health, their enthusiasm is not surprising.

The community-perceived leading barriers to improving local air quality were impact on the economy, lack of enforcement from government (federal and state), and a lack of research related to identifying the relationship between air quality and health. It was interesting to note the “other” comments, such as “apathy,” “malaise,” and a “feeling of helplessness.” These thoughts may be typical of underserved populations. Environmental groups, such as OCA and NCA, provide the opportunity for both increased understanding of environmental issues and engagement in solutions to community environmental problems.

The most trusted and perceived most knowledgeable source on risks of local industrial emissions was research scientists. Unfortunately, the community reported receiving very little information about these risks from research scientists. These findings highlight the current disconnect between researchers and community members while reinforcing the need for more researcher–community member interaction. Communicating science to lay audiences has always been a challenge (Bucchi and Neresini 2004; Condit 2004; Condit et al. 2004; Howel et al. 2003; Parrott et al. 2004; Popay and Williams 1996). Research scientists receive very little, if any, training in communicating their research findings to community members. Given that the community was very interested in learning more about the health risks of Mn exposure (87% responded that they wanting to know more), research scientists need to identify effective communication strategies to disseminate their findings into the communities where they conduct their research. It may be advantageous for research scientists to work with effective risk communication sources within communities, such as environmental groups, Web sites, and media. Our survey respondents did not include information sources, such as public health officials or schools, although we did include teachers. These may also be potential risk communication sources through which research scientists can effectively communicate their research findings.

Environmental groups were regarded as highly knowledgeable, highly trusted, and the leading source of information received about risks from local industrial emissions. We did not inquire further into which environmental groups (local or national) were perceived as such; however, both OCA and NCA were highly active in the community before and during the time of the survey, which probably contributed to this finding.

Respondents trust physicians as a source of information on risks associated with industrial emissions. This finding is consistent with others that the public has a high level of trust in clinicians to provide environmental health risk information, yet clinicians are often ill equipped to discuss environmental health risks with patients (Chai et al. 2001; Miller and Solomon 2003; Trasande et al. 2010; Wilson et al. 2000). Clinicians are uniquely positioned to address environmental health risks with vulnerable populations, yet there are limited educational tools for training clinicians in environmental health (Association of Occupational and Environmental Clinics 2011; ATSDR 2010), and many clinicians do not know where to access this information.

It is worth noting that the chemical industry officials are also viewed to be highly knowledgeable; however, they were also the least trusted information source.

The community-wide survey was conducted using a nonrandom sampling strategy. Our survey did not reach every community member, and every community member who received it may not have completed it, thus introducing nonresponse bias. In a study of 311 recruiter–applicant pairs, Goldberg (2003) found no difference in the outcome variables for the respondents compared with nonrespondents, so our survey is likely representative of the community.

*Academic–community partnership.* One of the ultimate goals was to develop a strong partnership between the academic research team institution and the community. One measure of the success of this goal is our codevelopment and award of two NIEHS-funded research projects: a CBPR award [Marietta Community Actively Researching Exposure Study (CARES)] and a PIR award described in “Materials and Methods.” Although an unmeasured outcome of our partnership that is possibly coincidental, it is worth noting that EMI, Inc. invited NCA for a face-to-face meeting a couple of weeks after the press release of our CBPR study award (February 2008). EMI, Inc. management now provides NCA with regular updates on their processes and emissions. Other potential influential factors for this change in approach with the community included the OCA–NCA Good Neighbor Campaign, which included writing letters to EMI, Inc. (Marietta, OH) management, posting yard signs throughout the community, and canvassing door-to-door; a change in management within EMI, Inc. in 2007; and negative press after the involvement of one of its employees in the first time a local citizen group (NCA) was banned from participating in the local Labor Day parade in 2007.

The NIEHS awards provided financial assistance to NCA and employment opportunities for the community. In the case of the PIR award, a portion of the grant was to be distributed directly to the community partner, in this case NCA. Although this proved challenging for a small group of citizens who did not have official nonprofit status, it did provide some independence related to project expenditures. Through the CBPR grant (Marietta CARES), we were able to provide several employment opportunities for community residents: one full-time position, seven part-time positions, and two positions for graduate students from Marietta College. Community members holding these positions received job-related training, such as neurodevelopmental testing, biological sample collection, study conduct, protection of human subjects, and home environmental sampling. Another training opportunity afforded through the PIR award consists of development of “science advocates,” a training program to enhance the scientific capacity of NCA members through workshops of their choice, such as “All about Modeling Air for Manganese,” “All about Air Sampling,” and “Air Particles and Health” (Ryan 2010). The latter workshop was developed into an online educational module for use by local journalists and community members. This and other educational information further describing all facets of the research study were incorporated into the study’s web site (http://www.eh.uc.edu/cares).

## Conclusion

Principles of community-based participatory research were a useful framework in building a strong academic–community partnership with an Appalachian-American community. The relationship with the community originated through a key community liaison. Through this liaison a community advisory board reflective of the community was developed and pilot research was conducted. Through these bidirectional preliminary research studies, community members were able to voice their thoughts and perceptions about their local air quality, express their willingness to participate in a research study before a large epidemiologic study took place, and identify their perception of environmental risk information sources. The community’s involvement in the research from its onset and throughout the research progress ensured that the research was relevant, was culturally appropriate, and will result in effective risk communication of the results.

In order to provide environmental health risk communication information to communities, research scientists should consider working with existing, effective risk communication sources within communities, such as environmental groups, Web sites, and media. Research is needed to identify which Web sites, environmental groups, and media outlets are most used and trusted by the community. Because the goal of environmental health research is to promote health and reduce the risk of disease across populations at highest risk, it is imperative that community needs and expertise are recognized and well integrated into the environmental health research protocols, and that academic researchers work collaboratively with community-based risk communication resources. This is particularly important when communities are high-risk populations, such as Appalachian Americans.
